# Experimental Investigation on the Influence of Water on Rockburst in Rock-like Material with Voids and Multiple Fractures

**DOI:** 10.3390/ma17122818

**Published:** 2024-06-10

**Authors:** Guokun Liu, Xiaohua Li, Zhili Peng, Wei Chen

**Affiliations:** 1 School of Building Engineering, Hunan Institute of Engineering, Xiangtan 411104, China; lixiaohuagcxy@163.com (X.L.); chenweiwade@mail.hnust.edu.cn (W.C.); 2 School of Resource, Environment and Safety Engineering, Hunan University of Science and Technology, Xiangtan 411201, China; chiguago@163.com; 3 Innovation Institute of Advanced Functional Materials, Hunan Institute of Engineering, Xiangtan 411104, China

**Keywords:** crack propagation, water, rockburst, damage mechanism, energy evolution

## Abstract

To investigate the influence of water content on the rockburst phenomena in tunnels with horizontal joints, experiments were conducted on simulated rock specimens exhibiting five distinct levels of water absorption. Real-time monitoring of the entire blasting process was facilitated through a high-speed camera system, while the microscopic structure of the rockburst debris was analyzed using scanning electron microscopy (SEM) and a particle size analyzer. The experimental findings revealed that under varying degrees of water absorption, the specimens experienced three stages: debris ejection; rockburst; and debris spalling. As water content increased gradually, the intensity of rockburst in the specimens was mitigated. This was substantiated by a decline in peak stress intensity, a decrease in elastic modulus, delayed manifestation of pre-peak stress drop, enhanced amplitude, diminished elastic potential energy, and augmented dissipation energy, resulting in an expanded angle of rockburst debris ejection. With increasing water content, the bond strength between micro-particles was attenuated, resulting in the disintegration of the bonding material. Deformation failure was defined by the expansion of minuscule pores, gradual propagation of micro-cracks, augmentation of fluffy fine particles, exacerbation of structural surface damage akin to a honeycomb structure, diminishment of particle diameter, and a notable increase in quantity. Furthermore, the augmentation of secondary cracks and shear cracks, coupled with the enlargement of spalling areas, signified the escalation of deformation failure. Simultaneously, the total mass of rockburst debris gradually diminished, accompanied by a corresponding decrease in the proportion of micro and fine particles within the debris.

## 1. Introduction

To meet the increasing energy demand, energy extraction is moving toward deeper levels, posing new challenges [1,2,3,4,5]. Rockburst, an abrupt and instantaneous engineering disaster, is triggered by deep construction, releasing energy along the free face, causing severe brittle damage and deformation [6,7,8]. These disasters threaten the safety of construction personnel, disrupt construction progress, and shorten equipment lifespan, posing a significant limitation to deep tunnel construction [9]. Extensive engineering research shows that rockbursts happen in arid regions, while natural rock masses have joint fissures [10,11,12]. Understanding the deformation and failure traits of such rock masses’ underwater influence is crucial [12,13,14,15,16].

When encountering rockburst phenomena in nature, researchers have often simulated actual working conditions in laboratory experiments to study rockburst mechanisms and inducing factors. Thus, laboratory simulation of rockburst experiments has become the mainstream approach for exploring rockburst issues. In recent years, scholars worldwide have conducted extensive research through laboratory experiments and numerical simulations, achieving significant results.

By conducting indoor rockburst experiments with prefabricated hole defects to simulate natural tunnel conditions, Gong et al. [17] concluded that during rock fracture and bursting, the damage zones on both sidewalls form two symmetrical V-shaped notches. The line connecting the centers of these notches is perpendicular to the direction of the maximum principal stress. In simulating rockburst using rock-like materials, Klammer et al. [18] showed that both stiffness and shape at the grain scale significantly influenced the intrinsic proneness to strainburst in rocks. Elongated grain shape, specific grain boundary characteristics, and a high stiffness contrast within the sample promote early crack initiation and propagation. Researching rockburst in fractured or faulted rock masses around deep tunnels [19,20], Amin Manouchehrian et al. [21] showed that the presence of a fault near a tunnel could induce rockburst if the fault was positioned and oriented in such a way that it promoted high stress and low-mine system stiffness. Researching the impact of moisture content on rockburst in rock masses [22], S Luo et al. [23] concluded that under uniaxial compression, the presence of water greatly degraded the peak strength, peak strain, and elastic modulus of the red sandstone. S Akdag et al. [24] and Chen et al. [25] conducted true triaxial experiments on various hard brittle rocks combined with SEM, discussing how differences in the intrinsic microstructure and fracture evolution in rocks were the primary factors leading to various rockbursts. Lin et al. [26] analyzed rockburst fragments using electron microscopy scanning, noting that damage from splitting mode failure was less severe than that from shear mode failure, with the primary mode of damage varying across different loading gradients.

The aforementioned results are abundant and highly representative, playing a crucial role in revealing the mechanisms and influencing factors of rockburst. However, existing research lacks studies on rockbursts in tunnels with varying moisture levels, especially in deep tunnels with natural fractures or defects caused by artificial excavation. Therefore, this study uses the Central Depression Zone of the Songliao Basin as a case study to prepare specimens with different water absorption levels, with prefabricated holes simulating deep tunnels and multiple prefabricated cracks simulating fractures around the rock mass. Uniaxial compression failure tests were conducted, and a high-speed camera system was employed to capture the rockburst process. Scanning electron microscopy and particle analyzers were utilized to analyze the microstructure of the rockburst. This study examined the uniaxial compressive strength, energy change trends, microstructural damage, crack propagation characteristics, and variation in rockburst fragments in single-hole specimens with multiple fractures under different water absorption levels. The results provide valuable insights for disaster prevention and mitigation in water-bearing deep tunnels with fractures.

## 2. Materials and Methods

### 2.1. Preparation of Rock Analog Samples

**Figure 1:**
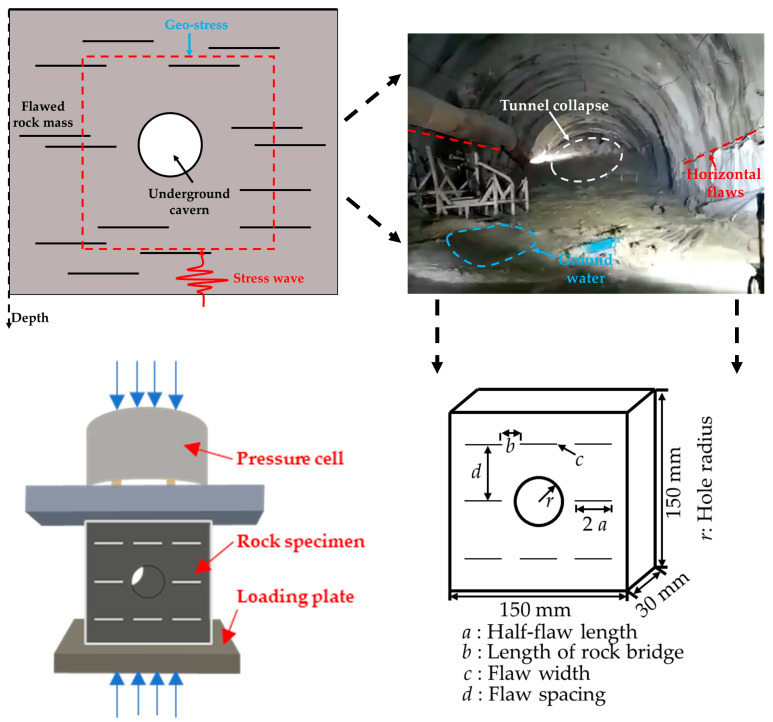
Indoor simulation of loading conditions for tunnels with groundwater and rock defects in underground engineering.

As illustrated in Figure 1, Schematic Diagram of the Specimen, the actual conditions are based on the collapse that occurred in the Q1 section of the tunnel in the ancient Longkeng Depression, located in the central Songliao Basin. Samples were prepared from typical weak sandy mudstone extracted from the Q1 section of the tunnel. Due to the presence of primary fractures and joints in deep hard rock formations, coupled with the difficulty in sampling, ensuring identical rock sample structures is challenging. Moreover, the prefabrication of fractures adds complexity. Therefore, rock analog materials with multiple pores and fractures are employed [27,28,29].

Preparation of Rock Analog Specimens: The mass proportions of raw materials are as follows: Ordinary Portland Cement (OPC) PO 52.5:Quartz Sand:Silica Powder:Distilled Water:Water Reducer:Defoamer = 10:8.5:1.2:2.8:0.15:0.15 [30]. After calculating and precisely weighing the raw materials, the mixture is stirred uniformly, and the concrete is poured into iron molds and leveled to form rectangular specimens measuring 150 mm × 150 mm × 30 mm. The vibrating table is then opened, and a scraper is used to smooth the surface, ensuring the removal of air bubbles while maintaining the flatness of the specimens. After 2 h of initial setting, prefabricated molds are inserted into the specimens, with circular holes of 40 mm diameter pre-made on the 150 mm × 150 mm face to simulate circular tunnels. Mica sheets with a thickness of 1 mm are inserted into prefabricated cracks with a width of 30 mm. The specimens are left to stand for 24 h before demolding after initial setting. After demolding, the specimens are placed in a curing chamber for 28 days to ensure maximum compressive strength. According to the International Society for Rock Mechanics standards, the deviation of adjacent surfaces’ verticality is within ±0.25°. Following the recommended methods of the International Society for Rock Mechanics, each group utilizes three specimens for repeated testing to reduce experimental errors [31].

**Figure 2:**
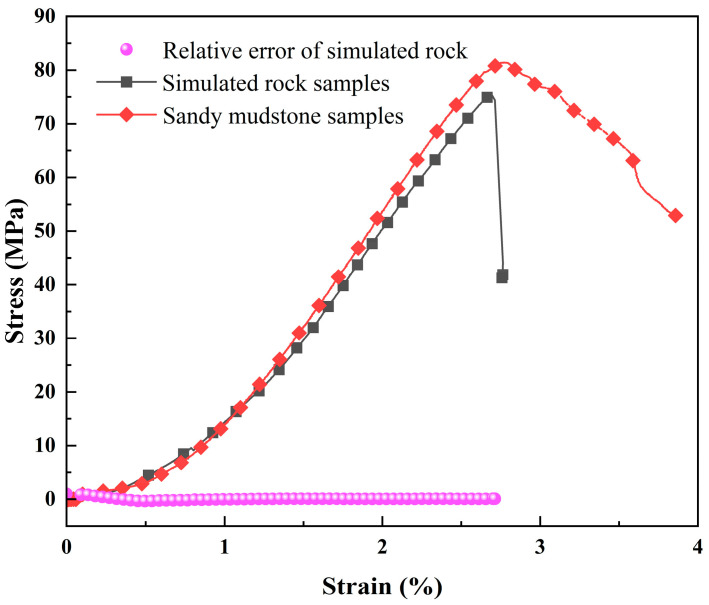
Comparison of experimental results between rock analog materials and original rock samples.

Prepare standard specimens for uniaxial compression testing to obtain the mechanical properties of rock analog materials and compare them with those of sandy mudstone (Figure 2). The stress–strain curves of rock-like materials and sandy mudstone are very similar. Table 1 compares four main parameters between rock-like materials and sandy mudstone. The composition of rock analog specimens appears reasonably appropriate.

To determine the time required to prepare rock analog specimens with different moisture contents, it is necessary to calibrate the water absorption curve before conducting the experiment. Initially, specimens are dried in a constant-temperature oven at 200 °C for 12 h to ensure complete loss of internal moisture. Subsequently, the specimens are immersed in ultrapure water, and regular measurements are taken and recorded. Experimental data analysis is conducted to plot the water absorption curve and calibrate it (using specimens labeled 1, 2, 3). The saturation water absorption rate of the specimens is determined to be 1.40%, with consistent measurements obtained after two hours [32,33,34,35]. The degree of water absorption is defined as the ratio of the water absorption rate at a certain time to the saturation water absorption rate, calculated using the following formula:

(1) $$P=\frac{\omega_{1}}{\omega_{0}\;}$$

where *P* represents the water saturation degree of the rock analog specimen [32,36,37]; *ω*_1_ denotes the water absorption rate at a certain time, and *ω*_0_ signifies the saturation water absorption rate of the rock analog specimen.

**Figure 3:**
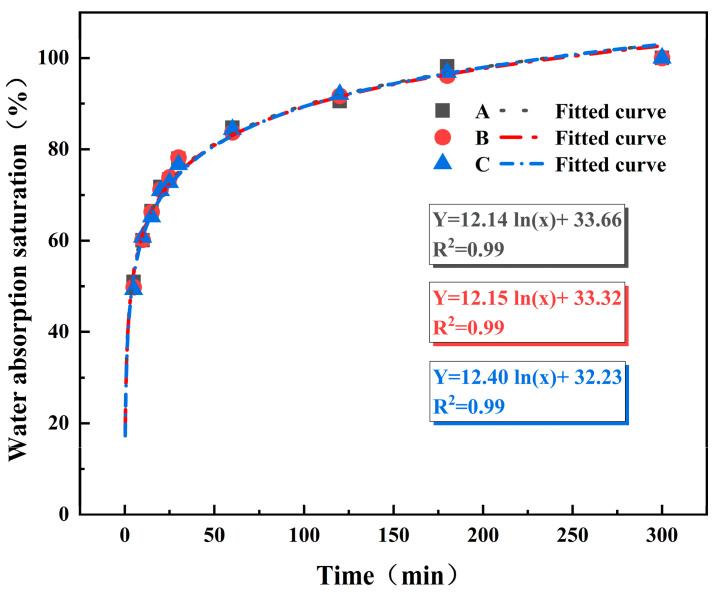
Curve of water absorption over time for simulated rock samples.

Figure 3 shows the water absorption calibration, with a gradient of 25%; rock analog specimens with water absorption degrees of 0%, 25%, 50%, 75%, and 100% are prepared. Combining the water absorption degree curve with actual measurement results, the required water absorption times for preparing specimens with water absorption degrees of 25%, 50%, 75%, and 100% are determined to be 21 s, 3.9 min, 44.3 min, and 515.2 min, respectively.

### 2.2. Experimental System

**Figure 4:**
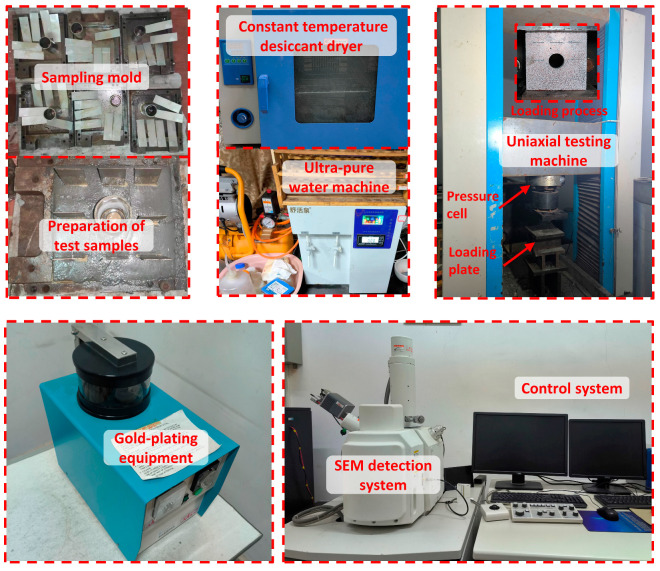
Rock-like specimen preparation process, experimental loading device, and SEM (Scanning Electron Microscope) detection system.

Figure 4 illustrates the specimen preparation process and the experimental loading system. The experimental setup utilizes the shear rheometer (RYL-600) produced by Changchun Chaoyang Instrument Co., Ltd. (Changchun, China) for uniaxial compression testing. It is employed for both uniaxial compression and transverse direct shear tests on rocks, as well as for uniaxial compression creep and transverse direct shear creep tests. These tests are capable of detecting the compressive strength and shear strength of rocks. Specimens are categorized into 5 gradients based on water absorption degree. Each group consists of 3 specimens to conduct repeated tests, aiming to reduce experimental errors and enhance accuracy. Prior to loading, a uniform coating of grease is applied to both the upper and lower loading surfaces of the specimens to minimize the influence of friction on the test results [38]. During the experiment, a consistent stress loading rate is set, with a displacement control velocity of 2 mm/min.

Throughout the loading process, an industrial camera focuses on the prefabricated holes of the specimens [39]. Real-time recording of the test process is conducted using an image sensor until the specimen reaches its peak value, upon which rockburst occurs, and recording is immediately halted.

SU3500 high resolution tungsten wire scanning electron microscope produced by Hitachi of Japan was adopted, specimens damaged by uniaxial loading were photographed at 500× and 1000× magnifications. According to the test platform specifications (Φ51 mm × 50 mm), samples with different water absorption levels were selected. Appropriately sized damaged fragments were screened for SEM analysis to identify microscopic deformation and damage characteristics. The fragments were cleaned with lint-free cloth and anhydrous ethanol, dried in an oven, and coated with a gold film before testing to enhance conductivity. This preparation ensured clear imaging of the microstructure for analysis and processing of representative data.

The Malvern laser particle size analyzer analyzes particles on microscopic damage structures. Debris from damaged specimens undergoes fractal statistical analysis to study how splashing characteristics vary with different water absorption levels.

## 3. Experimental Results and Discussion

### 3.1. Rockburst Energy Characteristics

**Figure 5:**
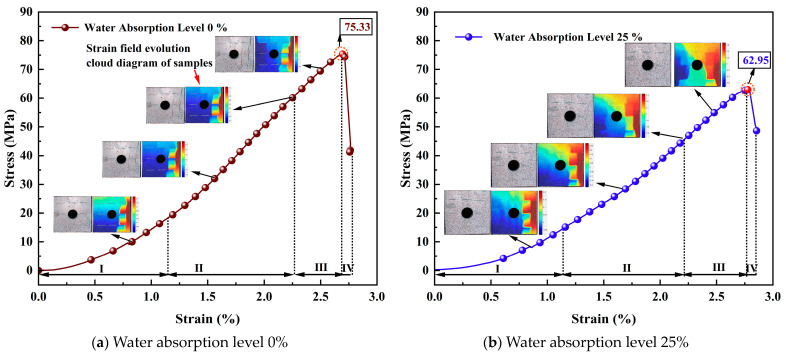

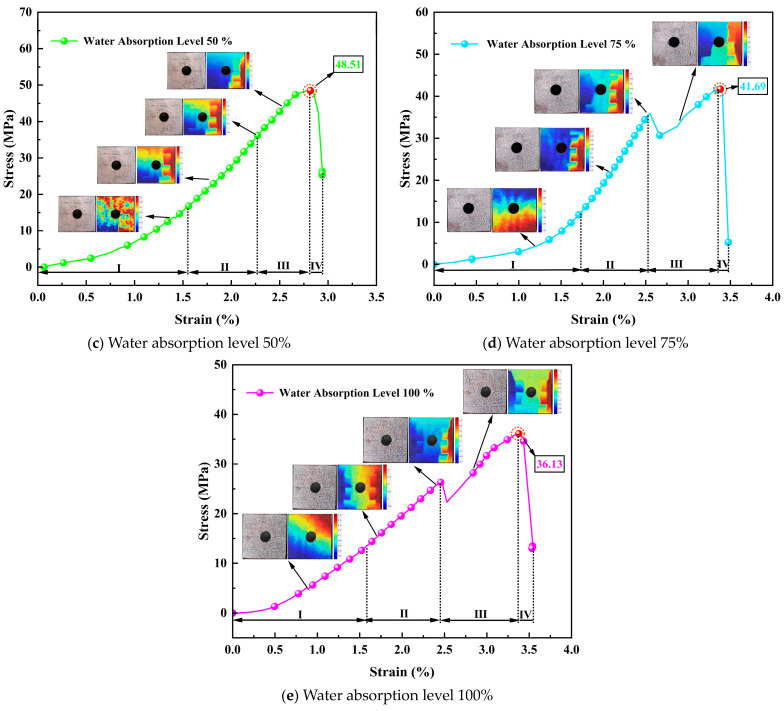
Different water absorption levels, typical stress–strain curves, and strain-field evolution cloud diagram of samples.

Figure 5 depicts the stress–strain curves and the evolution of strain field contours for samples with varying water absorption levels. The stress–strain curves undergo the following stages: compaction of pore and crack phase; elastic deformation stage; crack propagation stage; and strain softening stage [40].

**Figure 6:**
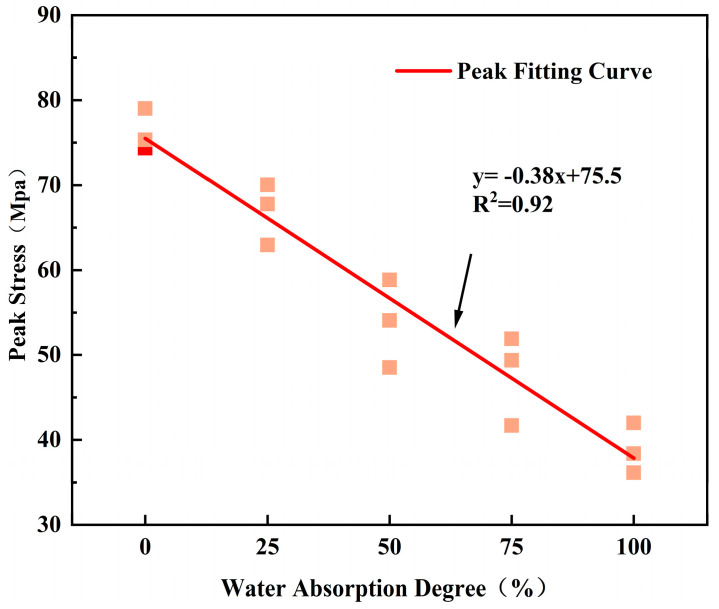
Trend diagram of peak stress variation with water content.

Stage I: Compaction of the pore and crack phase (Stage I) is characterized by a concave upward curve. Compaction closes the original micro-cracks and micro-pores of the samples. The length and degree of concavity in the upper concave section indicate rock porosity development. Dry samples undergo a shorter compaction stage, whereas strain significantly increases during the compaction stage of saturated samples. Increased water absorption and soaking time deepen internal dissolution within the samples. The slope gradually increases from an initial value of 0 to the elastic modulus (*E*_0_). The greater the water absorption degree, the deeper the concavity of the curve. There is no significant difference in the concavity between specimens with water absorption degrees of 0% and 25%. However, for specimens with water absorption degrees of 50%, 75%, and 100%, the compaction phase noticeably elongates;

Stage II: The elastic deformation stage (Stage II) is characterized by an approximately linear curve, where stress and strain are linearly related with a slope equal to the elastic modulus (*E*_0_). As shown in Figure 6, with the increasing gradient of water absorption degree, the elastic modulus gradually decreases, indicating a stronger effect of water erosion on the specimens. Compared to specimens with a water absorption degree of 0%, the elastic modulus decreases by 18%, 29.46%, 50.33%, and 62.19% for specimens with water absorption degrees of 25%, 50%, 75%, and 100%, respectively. This analysis suggests that an increase in water absorption degree reduces the elastic modulus of the specimen, diminishes its ability to resist deformation, and weakens the severity of rockburst;

Stage III: The crack propagation stage (Stage III) is characterized by a convex-upward curve. The slope gradually decreases from (*E*_0_) to 0, reaching the peak compressive strength when the slope becomes 0. When the stress curve reaches its peak value, accompanied by a “bang” sound, the rockburst phenomenon occurs simultaneously, and the stress curve rapidly declines. The influence of water absorption degree on the peak value is discussed in Figure 6. Prior to the peak, a stress drop occurs, indicating the development of internal cracks and pore connectivity within the specimen, but the strength of the specimen has not yet failed;

Stage IV: The strain softening stage (Stage IV) is characterized by an upward concave curve before point D (the inflection point after the peak) and a downward concave curve after point D, where the slope changes from 0 to negative.

Figure 6 illustrates the linear correlation between peak stress and water saturation degree under uniaxial compression for 15 specimens with different water saturation degrees. With the increase in water saturation degree, there is a linear decreasing trend in peak stress. Compared to fully dried specimens, the peak stress intensity decreases by 12.21%, 29.42%, 37.49%, and 49.06% for specimens with other water saturation degrees. The reduction in peak stress indicates that water has a softening effect on the rock analog specimens, leading to damage to the internal stress structure and weakening the severity of the rockburst.

To further investigate the influence of water absorption degree on the severity of rockburst from an energy perspective, an analysis of the energy dissipation relationship before the occurrence of rockburst will be conducted [41,42,43]. Assuming the specimen is an isolated closed system with no heat exchange with the surroundings, and the total input energy derived from external work is denoted as *U*, according to the first law of thermodynamics, we have the following equation:

(2) $$U=U^{e}+U^{d}$$

where *U^e^* represents the elastic strain energy that the rock can release after being subjected to external forces and represents the *U^d^* dissipated energy during the loading process of the rock.

**Figure 7:**
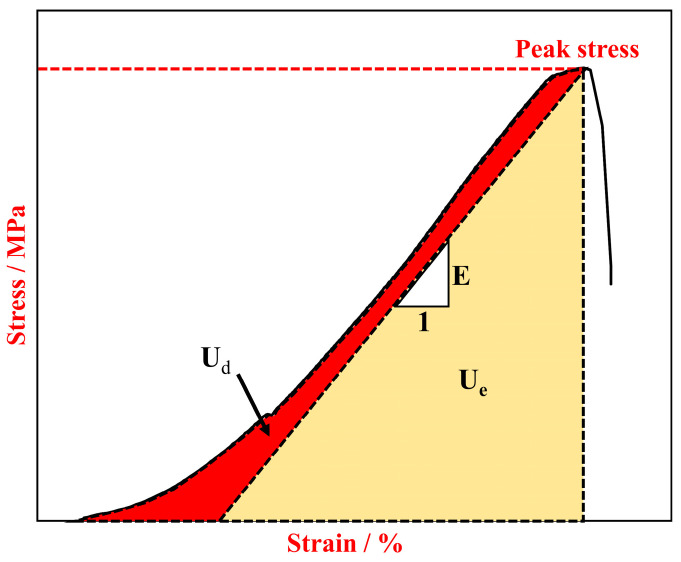
Schematic diagram of elastic energy and dissipation energy.

Figure 7 depicts the energy diagram of the stress–strain curve, illustrating the total input energy *U*, the dissipated energy *U^d^* during the loading process of the rock, and the elastic strain energy *U^e^* that the rock can release after being subjected to external forces. These energies can be calculated according to the following equations:

(3) $$U=\int_{0}^{\varepsilon }\sigma d\varepsilon$$

(4) $$U^{e}=\frac{1}{2}\sigma \varepsilon^{e}=\frac{\sigma^{2}}{2E_{0}}$$

(5) $$U^{d}=\int_{0}^{\varepsilon }\sigma d\varepsilon -\frac{\sigma^{2}}{2E_{0}}$$

**Figure 8:**
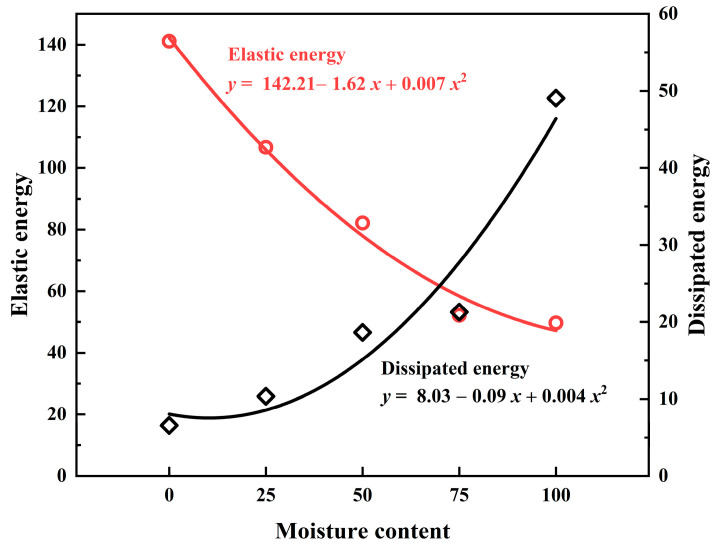
Influence of water content on elastic potential energy and dissipated energy.

Figure 8 shows the relationship between the elastic potential energy and the dissipated energy of the specimens for different water absorption levels. As the water absorption level increases, the elastic potential energy of the specimens gradually decreases, while the dissipated energy gradually increases.

The relationship between elastic energy and dissipated energy before rockburst for specimens with different degrees of water absorption is presented in Table 2. With the gradient increase in water absorption degree, the internal elastic strain energy gradually decreases, reducing by 24.40%, 41.80%, 63.08%, and 64.80%, respectively. Meanwhile, the dissipated energy gradually increases, with increments of 3.8, 12.1, 14.72, and 42.51 kJ·m^−3^. This indicates that the increase in water saturation degree leads to greater plastic deformation during the loading process of the rock analog specimens, weakening the ability of the specimens to store energy. The increase in porosity within the specimens leads to a reduction in elastic modulus and elastic strain energy, resulting in uneven stress distribution and the propagation and aggregation of microcracks within the specimens.

### 3.2. Rockburst Failure Process

**Figure 9:**
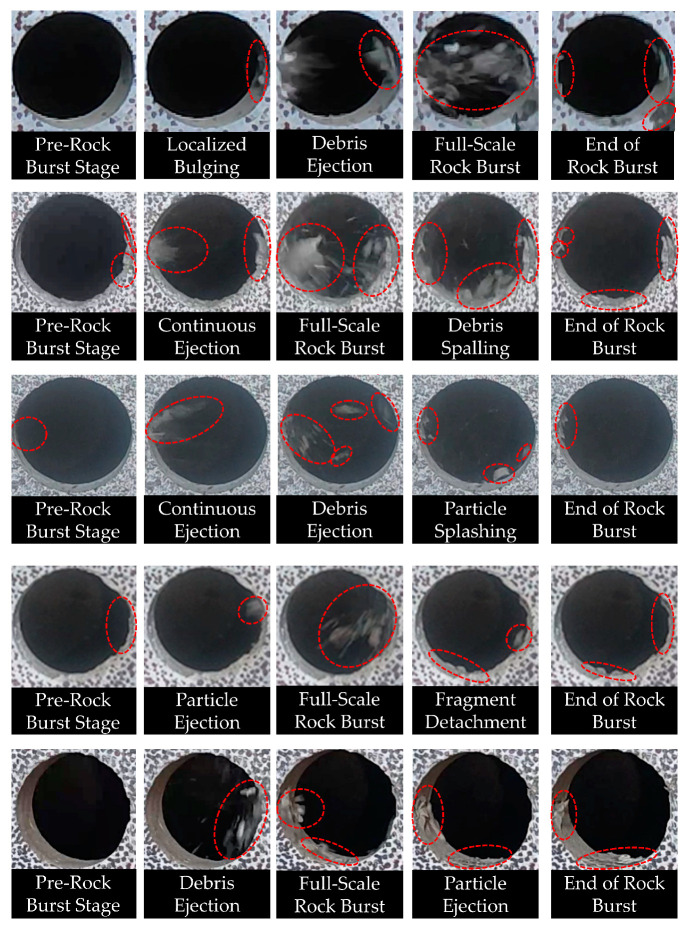
Different water absorption levels rockburst process diagram.

Figure 9 illustrates the rockburst damage process under uniaxial loading for specimens with different water absorption degrees. Specimens with varying water absorption degrees all undergo the following stages: pre-rockburst stage, localized bulging, particle ejection, full-scale rockburst, particle spalling, and end of rockburst [44,45].

Taking the specimen with 0% water saturation as an example, the rockburst process under uniaxial compression loading is described as follows:

During loading under displacement control at a rate of 2 mm/min, localized bulging and flake spalling occur on the right side of the specimen’s prefabricated hole. Compared to other stages, the spalled debris particles have larger diameters. As the test progresses, extensive particle ejection occurs on both sides of the prefabricated hole, with ejection angles close to 0°. The diameter of the ejected particles is significantly smaller compared to the particle spalling stage. Cracks appear on both sides of the hole, leading to extensive damage. During the full-scale rockburst stage, widespread particle splashing and spalling occur on the inner walls of both sides of the hole. A large amount of fine particle debris mixed with dust is ejected from both sides of the prefabricated hole, forming a blockage in the middle of the tunnel. The ejection of debris is intense. After the rockburst, significant damage is observed on both sides of the hole, with some debris falling from the inner walls. By comparing specimens with different water saturation degrees and observing the deformation and failure phenomena at different stages of rockburst, the following effects of water saturation on the rockburst damage mechanism can be inferred: as the water saturation of the specimens increases, the ejection angle of debris decreases, transitioning from horizontal ejection to parabolic ejection trajectories. The severity of damage to the prefabricated hole gradually decreases, and the ejection angle of particles increases with increasing water saturation degree.

With higher water saturation degrees, the number of ejected debris particles during a rockburst decreases. The ejected particles change from blocky with dust to blocky only, and their size increases gradually. The length and width of cracks generated in the prefabricated hole decrease with increasing water saturation degree. Both the phenomenon of debris ejection and damage to the prefabricated hole indicate the severity of rockburst, which decreases with increasing water saturation degree in the rock analog specimens.

### 3.3. Microscopic Characteristics of Rockburst

Microscopic structural damage exists within specimens with different degrees of water absorption. As the water absorption degree increases gradually from 0% to 100%, it can be clearly observed from SEM images that the damaged surfaces transition from relatively smooth to fractured and uneven states, and there is a noticeable change in the diameter of the peeled-off particles due to compression. Gaps between particles gradually develop, with particle structures being denser at low water saturation degrees and gradually fracturing as the water absorption degree increases. Eventually, flocculent particles appear, and the binding material between particles decreases, leading to a looser structure. The number and size of pores increase gradually, indicating the damage pattern of the microscopic structure after stress-induced damage to the specimens.

**Figure 10:**
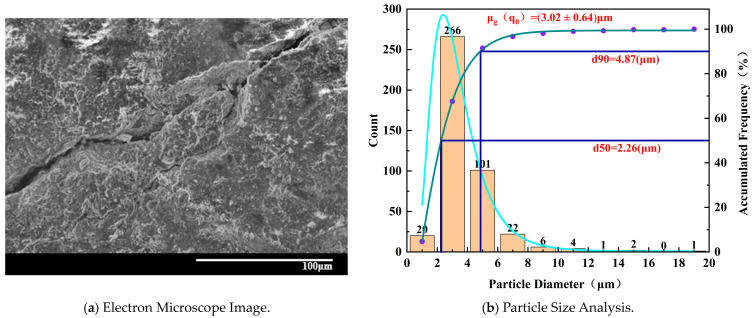
Microscopic characteristics of 0% water absorption degree specimen.

Figure 10 shows that for the specimen with 0% water absorption, the electron microscope image reveals a fractured structure with significant cracks. The interconnected cracks exhibit shallow depths, and the peeled-off damaged particles are relatively large, maintaining overall integrity and regular shape. The particles are densely arranged and interconnected without significant pores observed. The distribution of pores is low. The scattered particles mainly consist of larger block-like particles, and there is tight binding between the particles without the presence of flocculent particles. Although there are smaller particles, their quantity is relatively small. Microscopic pores are observed along the crack regions, with particle sizes decreasing gradually from large to small.

**Figure 11:**
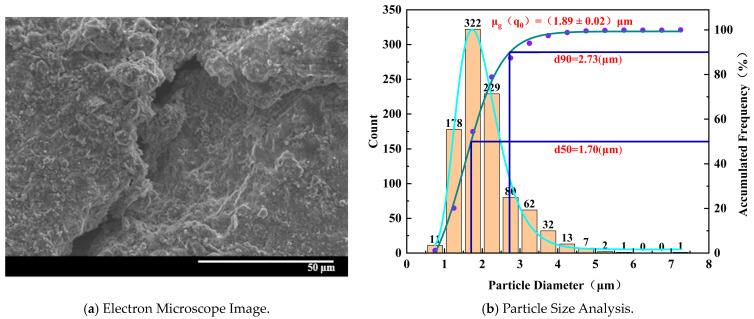
Microscopic characteristics of 25% water absorption degree specimen.

Figure 11 shows that for the specimen with 25% water absorption, the electron microscope image depicts a more fragmented structure with multiple instances of peeling and larger voids. The density of distribution of tiny particles is higher, and the overall particle diameter decreases. The proportion of larger particles with regular shapes is smaller. Microscopic pores appear more frequently between the particles, and the gaps between particles become more apparent, indicating a progressively looser arrangement of particles. Smaller flocculent particles are observed, suggesting damage to the binding material due to water erosion, resulting in irregular flocculent particles.

**Figure 12:**
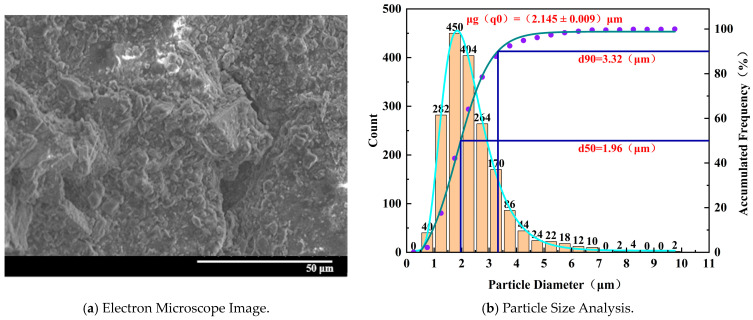
Microscopic characteristics of 50% water absorption degree specimen.

Figure 12 demonstrates that for the specimen with 50% water absorption, the electron microscope image shows an overall fragmented and loose structure, resembling a honeycomb pattern. The cracks are interconnected and well-developed, with deeper depths. The larger-diameter particles on the structure surface are significantly reduced, and clear gaps appear between particles. The damaged particles exhibit irregular shapes and breakdowns, with a noticeable increase in flocculent particles and a higher proportion of small particle distribution.

**Figure 13:**
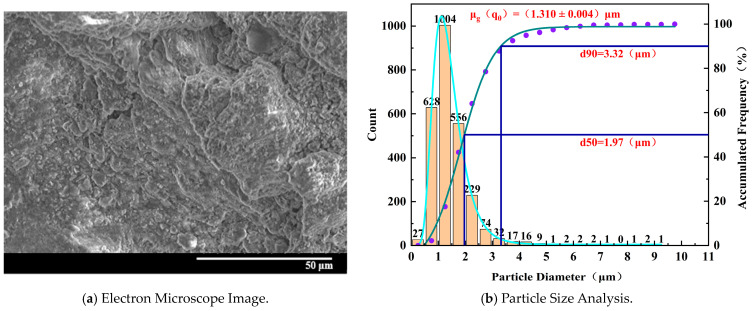
Microscopic characteristics of 75% water absorption degree specimen.

Figure 13 demonstrates that for the specimen with 75% water absorption, the electron microscope image shows a transition from a relatively fragmented structure to a honeycomb-like structure with severe fragmentation. Microscopic pores between particles gradually connect to form distinct seams, and the number of cracks increases significantly. The pores enlarge, transforming from particle-bound structures to isolated distributions. The edges of microscopic particles on the structure surface become curled, forming numerous flocculent particles. The overall particle diameter on the fractured structure surface notably decreases, and the layered structure gradually disintegrates into tiny particles.

**Figure 14:**
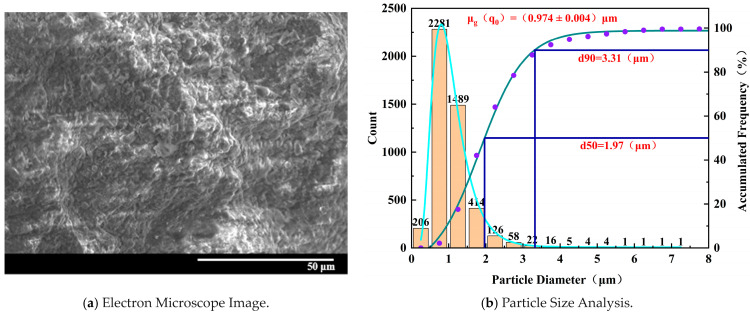
Microscopic Characteristics of 100% Water Absorption Degree Specimen.

Figure 14 shows that for the specimen with 100% water absorption, the electron microscope image reveals a honeycomb-like structure with extensive fragmentation. Numerous irregular voids caused by peeling are evident, and the scattered particles are primarily tiny. The voids between particles are completely interconnected, forming micro-cracks, and the micro-pores develop into larger voids. The binding particle structure disintegrates, resulting in numerous flocculent particles. Compared to the specimen with 0% water absorption, significant changes in the microstructure are observed, including a notable decrease in overall particle diameter, a looser particle structure, a significant increase in pore/crack formation, and a transition from a compact structure to a honeycomb-like one. The appearance of numerous flocculent particles indicates significant damage due to water erosion.

To analyze the particle size distribution of samples with different water absorption levels, we sampled at various locations on the specimens to account for positional and characteristic variations. The results were then averaged to minimize random variability and ensure experimental reliability. Based on the normalized distribution of particle sizes on typical damaged surfaces of samples with different water absorption levels, it can be observed that as water absorption increases, the average particle size of microscopic particles gradually decreases. The overall slope of the normal distribution curve significantly increases, the particle diameter significantly shifts to the left, and the overall number of particles increases. This indicates that as water absorption increases, microstructural damage becomes more severe.

Summarizing the microstructural changes with varying degrees of water absorption, the dissolution and erosion of cementitious materials, along with the physical and chemical effects of water, lead to the development and interconnection of micro-pores and cracks in the rock-like material [46,47]. These structures become uniform in size; the particle structure gradually loosens and breaks, and the particle diameter decreases, resulting in the deterioration of the macroscopic strength of the rock-like material.

### 3.4. Crack Propagation Characteristics

**Figure 15:**
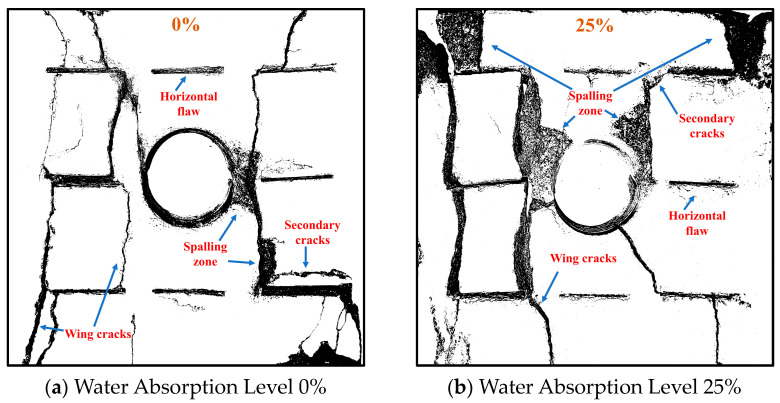

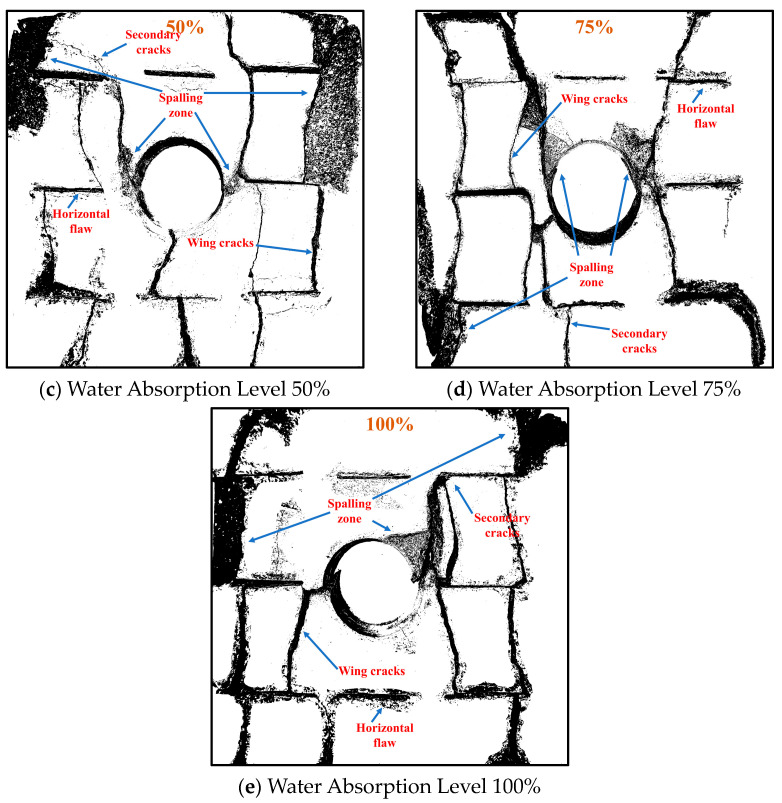
Crack Propagation Diagram at Various Levels of Water Absorption.

The fracture patterns on the front and back sides of the specimens are essentially identical; thus, only the fracture patterns on the front side are analyzed. Figure 15 illustrates the complete fracture propagation patterns of specimens with varying degrees of water absorption [48,49,50,51,52]. Enhanced water absorption results in increased dissipation energy within the samples. Due to the effect of dissipation energy, the propagation of microcracks within the samples markedly diminishes the energy storage capacity, with elastic energy decreasing by 24.40%, 41.80%, 63.08%, and 64.80%, respectively. The substantial reduction in elastic energy results in increased macroscopic damage.

Analysis of the fracture propagation patterns reveals that cracks primarily originate from both ends of the horizontally pre-existing cracks [53], forming wing cracks that penetrate through the rock bridge. Simultaneously, tension cracks develop on both sides of the holes. Moreover, during the loading process, rockbursts occur, leading to debris ejection and stripping on both sides of the pre-existing holes. The V-shaped damage caused by debris ejection intersects with secondary shear cracks and wing cracks, forming main and sub-diagonal penetrating cracks. As water absorption increases, the number of wing cracks gradually increases, and secondary shear cracks penetrate the pre-existing holes from both sides of the cracks. Additional debris damage occurs on both sides of the specimen’s diagonal line. Thus, with the increase in water absorption, the deformation and damage severity of the specimens gradually intensify.

### 3.5. Characteristics of Rockburst Debris

**Figure 16:**
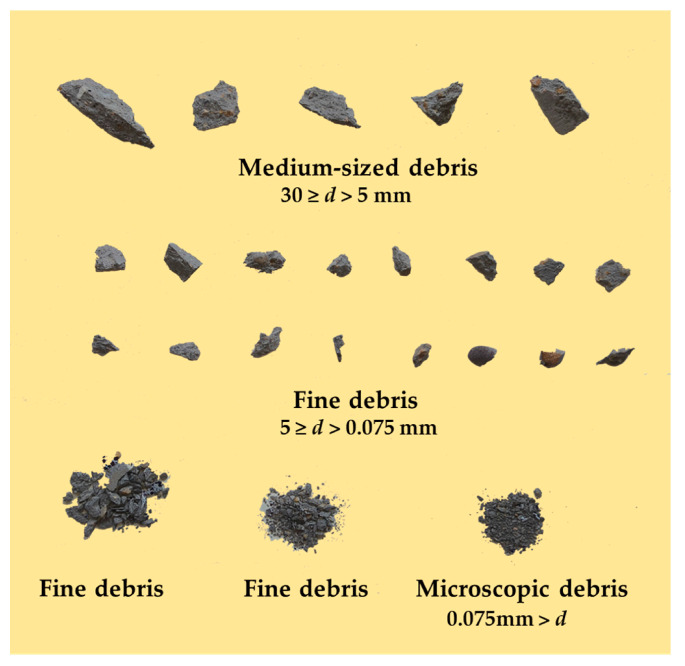
Typical Fractal Distribution Diagram of Detrital Particles.

Debris fragments following a rockburst can intuitively reflect the extent of damage and characteristics of the rock under uniaxial conditions [13,54,55]. The debris collected after the experiment is processed by sieving with calipers and a sieve. Figure 16 presents a schematic diagram of typical rockburst debris.

**Figure 17:**
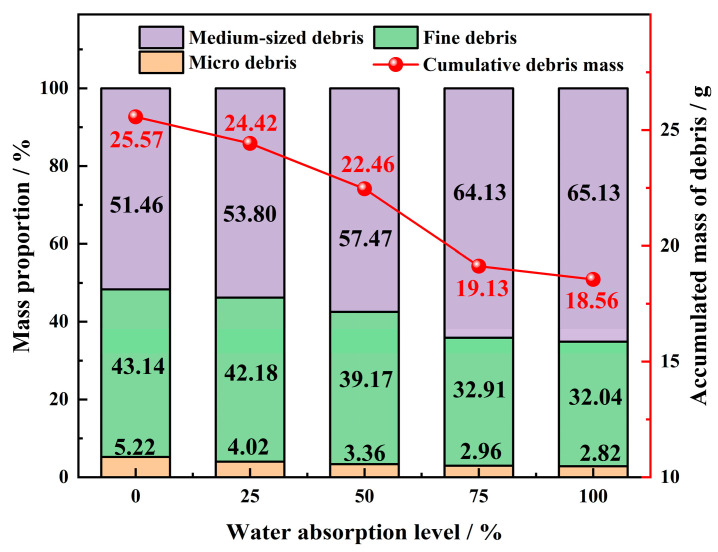
Mass distribution characteristics of fragments with varying water content.

Based on the diameter of the debris, it can be classified into microscopic, fine, and medium debris. Mass statistics are conducted based on the particle size of the debris, and Figure 17 shows the mass percentage of medium, fine, and microscopic debris.

Plotting the proportion of fine, medium, and microscopic debris generated by specimens with different degrees of water absorption after stress loading. From Figure 17, it is evident that as the water absorption degree increases, the total mass of debris generated by the rockburst decreases. The mass percentage of fine and microscopic debris in simulated rock samples gradually decreases, while the mass percentage of medium-sized debris shows an increasing trend. The decrease in the proportion of fine and microscopic debris indicates that the particle diameter of debris generated by rockbursts is larger. The proportion of medium-sized debris gradually increases, indicating the presence of pore water pressure within the internal pores of the specimen, which reduces the strength of the specimen. The above analysis results indicate that the increase in water absorption degree leads to a gradual increase in the diameter of debris particles, a decrease in fine and microscopic debris particles, a decrease in the energy released by rockbursts, and a weakening of the intensity of rockbursts.

## 4. Conclusions

In this study, uniaxial compression tests, a high-speed camera system, SEM tests, and particle size analysis were used to conduct simulated rockburst experiments on multi-defect specimens with varying water absorption levels. The impact of water on rockburst in multi-defect specimens was comprehensively analyzed. The main conclusions of this study are as follows:

1. The rockburst damage process remains consistent across different water absorption levels. Higher water absorption results in decreased mass of rockburst debris, reduced proportion of fine particles, lower elastic potential energy, and increased dissipation energy, resulting in reduced severity of rockburst;
2. Increasing water absorption reduces the diameter of microscopic structural particles, increases their quantity, and fragments the structure. Additionally, it leads to more wing cracks and secondary cracks penetrating pores, forming diagonal damage patterns. This reduces specimen energy storage capacity and rockburst severity.

## Figures and Tables

**Table 1:** Comparison of parameters between standard samples of artificial rock and sandy mudstone.

Types of Samples	Uniaxial Compressive Strength MPa	Elastic Properties U_e_/(kJ·m^−3^)	Poisson’s Ratio	Saturation Water Absorption Rate %
Rock-like material	75.34	68.92	0.23	1.4
Sandy mudstone	81.42	78.97	0.21	2.3

**Table 2:** Relationship between Elastic Energy and Dissipation Energy of Specimens with Different Water Absorption Degrees before Rockburst.

Water Absorption Level/%	Number	Elastic Properties U_e_/(kJ·m^−3^)	Mean Value /(kJ·m^−3^)	Dissipation Energy U_d_/(kJ·m^−3^)	Mean Value /(kJ·m^−3^)
0%	1	185.37	141.13	4.89	6.55
	2	113.57		6.35	
	3	124.46		8.42	
25%	4	102.55	106.69	1.17	10.35
	5	100.03		25.53	
	6	117.49		4.34	
50%	7	103.83	82.14	12.41	18.65
	8	84.10		31.78	
	9	58.49		11.75	
75%	10	42.86	52.11	39.05	21.27
	11	54.82		12.38	
	12	58.66		12.39	
100%	13	49.18	49.68	29.74	49.06
	14	40.28		21.23	
	15	59.60		96.22	

## Data Availability

The data are not publicly available due to privacy.
